# CTLA4 blockade increases Th17 cells in patients with metastatic melanoma

**DOI:** 10.1186/1479-5876-7-35

**Published:** 2009-05-20

**Authors:** Erika von Euw, Thinle Chodon, Narsis Attar, Jason Jalil, Richard C Koya, Begonya Comin-Anduix, Antoni Ribas

**Affiliations:** 1 Department of Surgery, Division of Surgical Oncology, University of California, Los Angeles (UCLA), Los Angeles, California, USA; 2 Department of Medicine, Division of Hematology/Oncology, UCLA, Los Angeles, California, USA; 3 Jonsson Comprehensive Cancer Center, UCLA, Los Angeles, California, USA

## Abstract

**Background:**

Th17 cells are CD4+ cells that produce interleukin 17 (IL-17) and are potent inducers of tissue inflammation and autoimmunity. We studied the levels of this T cell subset in peripheral blood of patients treated with the anti-CTLA4 antibody tremelimumab since its major dose limiting toxicities are inflammatory and autoimmune in nature.

**Methods:**

Peripheral blood mononuclear cells (PBMC) were collected before and after receiving tremelimumab within two clinical trials, one with tremelimumab alone (21 patients) and another together with autologous dendritic cells (DC) pulsed with the melanoma epitope MART-1_26–35 _(6 patients). Cytokines were quantified directly in plasma from patients and after *in vitro *stimulation of PBMC. We also quantified IL-17 cytokine-producing cells by intracellular cytokine staining (ICS).

**Results:**

There were no significant changes in 13 assayed cytokines, including IL-17, when analyzing plasma samples obtained from patients before and after administration of tremelimumab. However, when PBMC were activated *in vitro*, IL-17 cytokine in cell culture supernatant and Th17 cells, detected as IL-17-producing CD4 cells by ICS, significantly increased in post-dosing samples. There were no differences in the levels of Th17 cells between patients with or without an objective tumor response, but samples from patients with inflammatory and autoimmune toxicities during the first cycle of therapy had a significant increase in Th17 cells.

**Conclusion:**

The anti-CTLA4 blocking antibody tremelimumab increases Th17 cells in peripheral blood of patients with metastatic melanoma. The relation between increases in Th17 cells and severe autoimmune toxicity after CTLA4 blockade may provide insights into the pathogenesis of anti-CTLA4-induced toxicities.

**Trial Registration:**

**Clinical trial registration numbers**: NCT0090896 and NCT00471887

## Introduction

Monoclonal antibodies blocking the cytotoxic T lymphocyte associated antigen 4 (CTLA4), a key negative regulator of the immune system, induce regression of tumors in mice and humans, and are being pursued as treatment for cancer [1-4]. CTLA4 blocking antibodies break tolerance to self tissues, as clearly demonstrated by the autoimmune phenomena in CTLA4 knock out mice [5,6], which results in autoimmune toxicities in patients. Understanding the immunological mechanisms guiding antitumor responses and anti-self toxicities may allow improving the use of this class of agents in the clinic.

The emerging clinical data suggests that a minority of patients with metastatic melanoma (in the range of 10%) achieve durable objective tumor responses when treated with CTLA4 blocking monoclonal antibodies, with most being relapse-free up to 7 years later. However, a significant proportion of patients (in the range of 20–30%) develop clinically-relevant toxicities, most often autoimmune or inflammatory in nature [2-4]. There is a prevalent thought that toxicity and response are correlated after therapy with anti-CTLA4 blocking monoclonal antibodies. This conclusion is based mainly on statistical correlations in 2 × 2 tables grouping patients with toxicities and/or objective responses. However, even though patients with a response are more likely to have toxicities in these series, most patients with toxicity do not have a tumor response and there are occasional patients with an objective tumor response who never developed clinically-relevant toxicities [2,7], thereby suggesting to us that the relationship between toxicity and response is not linear. If we assume that both phenomena (toxicity and response) are mediated by activation of lymphocytes, then we need to question their antigen specificity, since it is unlikely that the same T cells that mediate toxicity in the gut, for example, will be responsible for antitumor activity against melanoma. It is more likely that the same threshold of CTLA4 blockade may lead to activation of lymphocytes reactive to self-tissues and cancer. Therefore, we studied a differentiated subset of cells termed Th17, which have emerged as key mediators of autoimmunity and inflammation for their potential implication in toxicity and responses after anti-CTLA4 therapy.

The description of Th17 cells has substantially advanced our understanding of T cell-mediated inflammation and immunity [8]. These cells are characterized as preferential producers of IL-17A (also known as IL-17), IL-17F, IL-21, IL-22, and IL-26 in humans. The production of IL-17 is used to identify Th17 cells and differentiate them from IFN-γ-producing Th1 cells, or IL-4-producing Th2 cells. The transcription factor retinoic-acid-related orphan receptor-γτ (ROR-γτ) and IL-1β and IL-23 are important for the generation of human Th17 cells *in vitro *and *in vivo *[8,9]. Th17 cells are potent inducers of tissue inflammation, and dysregulated expression of IL-17 appears to initiate organ-specific autoimmunity; this has been best characterized in mouse models of colitis [10], experimental autoimmune encephalomyelitis (EAE) [11,12], rheumatoid arthritis [13] and autoimmune myocarditis [14]. In these models, mice treated with anti-IL-17 antibodies have lower incidence of disease, slower progression of disease and reduced scores of disease severity. Treatment with anti-IL-17 antibodies nine days after inducing EAE significantly delayed the onset of paralysis. When the treatment was started at the peak of paralysis, disease progression was attenuated [15]. Cytokines like IL-17A and IL-17F, as well as IL-22 (a member of the IL-10 family) are produced by Th17 and evoke inflammation largely by stimulating fibroblasts, endothelial cells, epithelial cells and macrophages to produce chemokines, cytokines and matrix metalloproteinases (MMP), with the subsequent recruitment of polymorphonuclear leukocytes to sites of inflammation [16]. In addition, Th17 cells have been associated with effective tumor immunity in a model of adoptive transfer of TCR transgenic CD4+ T cells specific for the shared self-tumor antigen tyrosinase-related protein 1 (TRP1) [17]. These cells were used for the treatment of the poorly immunogenic B16 murine melanoma, and the therapeutic efficacy of Th1, Th17, and Th0 CD4+ T cell subsets was studied. The investigators demonstrated that the tumor-eradicating population was the Th17 cells [17].

Tremelimumab is a fully human IgG2 monoclonal antibody with high binding affinity for human CTLA-4 [18]. This antibody is in late stages of clinical development in patients with metastatic melanoma [3,4,19]. It has a long plasma half life of 22 days, which is identical to the half life of endogenous IgG2s. When administered at doses of 10 to 15 mg/kg, plasma levels of tremelimumab beyond 30 μg/ml are achievable for 1 to 3 months [19]. This sustained antibody concentration in plasma correlates with the *in vitro *concentrations required to have a biological effect of CTLA4 blockade [18], suggesting that sustained therapeutic levels of this antibody can be achieved with the doses administered to patients. The remarkably durable antitumor activity of tremelimumab in a small subset of patients is mediated by T cell-induced tumor regressions [20], but its use is limited by autoimmune and inflammatory toxicities [3,4]. Therefore, understanding the mechanisms that lead to toxicity and antitumor response are of great importance to the development of CTLA4 blocking antibodies. Here we report the increase in Th17 cells in patients with metastatic melanoma after treatment with tremelimumab with or without DC vaccines, and its preferential increase in patients that develop clinically-relevant inflammatory and autoimmune toxicities.

## Patients and methods

### Description of Clinical Trials

Peripheral blood samples were obtained from leukapheresis procedures from 27 patients with metastatic melanoma that had been treated at UCLA in two investigator-initiated research protocols that included the anti-CTLA4 blocking antibody tremelimumab (Pfizer, New London, CT). In both clinical trials, patients underwent pre- and post-dosing apheresis collecting PBMC and plasma, and the UCLA IRB approved informed consent forms described their banking for immune monitoring assays. Six patients were treated in a phase I clinical trial of three biweekly intradermal (i.d.) administrations (study days 1, 14 and 28) of a fixed dose of 1 × 10^7 ^autologous DC pulsed with the MART-1_26–35 _immunodominant peptide epitope (MART-1_26–35_/DC) manufactured as previously described [21], concomitantly with a dose escalation of tremelimumab at 10 (3 patients) and 15 mg/kg (3 other patients) every 3 months (UCLA IRB# 03-12-023, IND# 11579, Trial Registration number NCT0090896). The samples from these patients were coded with the study denomination of NRA and a patient-specific number. The remaining 21 patients were enrolled in a phase II clinical trial of single agent tremelimumab (UCLA IRB# 06-06-093, IND# 100453, Trial Registration number NCT00471887) administered at 15 mg/kg every 3 months. The samples from these patients were coded with the study denomination of GA and a patient-specific number. Objective clinical responses were recorded following a slightly modified Response Evaluation Criteria in Solid Tumors (RECIST) [22]. The modification was to consider measurable disease lesions in the skin and subcutaneous lesions detectable by physical exam, but not by imaging exams, if they were adequately recorded at baseline using a camera with a measuring tape or ruler. Toxicities were recorded during the first 3 months of therapy (one cycle of tremelimumab-based therapy), since the post-dosing leukapheresis was performed only during the first cycle of therapy, most frequently between 30 and 60 days from the first dose of tremelimumab. The post-dosing leukapheresis were performed a median of 41 days after the dose of tremelimumab (range 28 to 81, with 6 cases out of the 30–60 day range). In all cases, concentrations of tremelimumab in peripheral blood should have been above 10 μg/ml at the time of cell harvesting by leukapheresis, which is the minimum concentration of tremelimumab that stimulated a biological effect consistent with CTLA4 blockade in preclinical studies [18]. Adverse events attributed to tremelimumab by the study investigators were graded according to the NCI common toxicity criteria version 2.0 [23]. Dose limiting toxicities (DLTs) were prospectively defined in both studies as any treatment-related toxicity equal or greater than grade 3, or the clinical evidence of grade 2 or higher autoimmune reaction in critical organs (heart, lung, kidney, bowel, bone marrow, musculoskeletal, central nervous system and the eye).

### Sample Procurement and Processing

PBMC were collected from patients receiving tremelimumab-containing experimental immunotherapy by a leukapheresis procedure. Leukaphereses were planned as part of the pre-dosing procedures, and one to two months after receiving the first dose. Leukapheresis products were used to isolate PBMC by Ficoll-Hypaque (Amersham Pharmacia, Piscataway, New Jersey, USA) gradient centrifugation. PBMC were cryopreserved in liquid nitrogen in Roswell Park Memorial Institute medium (RPMI, Gibco-BRL, Gaithersburg, Maryland, USA) supplemented with 20% (all percentages represent v/v) heat-inactivated human AB serum (Omega Scientific, Tarzana, California, USA) and 10% dimethylsulfoxide (Sigma, St. Louis, Missouri, USA). One hundred milliliters of plasma were collected during the same apheresis procedures and were frozen at -20°C in 1 to 10 ml single use aliquots. Plasma samples were thawed and used immediately to measure cytokines.

### Cytokine Detection in Plasma

Plasma samples from patients enrolled in the GA study were assessed for 12 cytokines using a cytokine suspension array detection system. The cytokines quantified were IL-1β, IL-2, IL-4, IL-5, IL-6, IL-10, IL-12 (p70), IL-13, tumor necrosis factor alpha (TNF-α), IFN-γ, granulocyte colony-stimulating factor (G-CSF), monocyte chemoattractant protein 1 (MCP-1/MCAF) and Chemokine (C-C motif) ligand 5, CCL-5 (RANTES). The assay was done according to the manufacturer's instructions in 96-well plates (Millipore, Billerica, Massachusetts, USA). Samples were analyzed using the Bio-Plex suspension array system (Bio-Rad Laboratories, Hercules, California, USA) and the Bio-Plex manager software with 5PL curve fitting. In addition, IL-17, a cytokine not represented in the multiplex cytokine detection kit described above, was quantified in plasma using a commercially available ELISA according to the manufacturer's instructions (eBioscience, San Diego, California, USA). Cytokine concentrations were analyzed in neat (undiluted) samples. The ranges of detection were from 6.9 to 5000 pg/ml for IL-4, IL-5, IL-6, IL-10, IL-13, TNF-α, from 12.3 to 9000 pg/ml for INF-γ and MCP-1, from 4.1 to 3000 pg/ml for RANTES and from 3.9 to 500 pg/ml for IL-17.

### Cytokine Detection in Culture Supernatants

Cryopreserved PBMC aliquots collected before and after administration of tremelimumab within the GA and NRA studies were thawed and immediately diluted with RPMI complete media consisting of 10% human AB serum and 1% penicillin, streptomycin, and amphotericin (Omega Scientific). Cells were washed and subjected to enzymatic treatment with DNAse (0.002%, Sigma) for 1 hour at 37°C. Cells were washed again, and an aliquot of each sample was sorted using CD4+ magnetic cell sorting beads following the manufacturer's instructions (Miltenyi Biotec Inc., Auburn, California, USA). 2 × 10^6 ^pre- and post-dosing PBMC, and the same number of magnetic colum-sorted CD4+ cells, were incubated for 4 days with 50 ng/ml of anti-CD3 (OKT3, Ortho-Biotech, Bridgewater, New Jersey, USA) and 1 μg/ml of anti-CD28 (BD Biosciences, San Diego, California, USA) in 6-well plates. Cells were spun down, and the supernatants were collected for IL-17 by ELISA assay. All samples were measured in duplicates.

### Intracellular Flow Cytometry for IL-17

To enumerate Th17 cells by ICS, PBMC or sorted CD4+ cells were activated as described above for 4 days in anti-CD3 and anti-CD28, and then re-stimulated for 5 hours with 5 μg/μl PMA and 5 μg/μl ionomycin in the presence of 1 μl/ml of a protein transport inhibitor containing brefeldin A (GolgiPlug, BD Biosciences) in FACS tubes. Cells were then surface stained with phycoerythrin (PE) anti-human CD4 and peridinin-chlorophyll-protein complex (PerCP) anti-CD3 (BD Biosciences) at room temperature for 15 minutes, permeabilized and then stained intracellularly with APC anti-IL-17 according to the manufacturer's instructions (eBioscience). Isotype antibody controls were used to enable correct compensation and to confirm antibody specificity. Flow cytometry analysis was conducted using FACSCalibur (BD Biosciences), and the data was analyzed using FlowJo software (Tree Star, Inc., San Carlos, California, USA).

### Statistical analysis

Statistically significant differences in the concentration or percentage of IL-17 cytokine and Th17 cells between pre- and post-treatment samples were analyzed using a two-sided Student's paired t test using the Prism package (GraphPad Software, Inc., San Diego, California, USA). For all statistical analysis, the p value was set at p < 0.05. There was no correction for multiple comparisons, and all statistical analysis should be considered exploratory. All error bars shown in this paper are standard errors of the means (SEM).

## Results

### Patient Characteristics, Response and Toxicity

Table 1 provides a description of the study patients, their baseline characteristics, the treatment received and the outcome after therapy. Two thirds of the patients had M1c metastatic melanoma (visceral metastasis and/or high LDH), and most of the remaining patients had either in transit (stage IIIc) or soft tissue and nodal metastasis (M1a). There were 6 patients with objective tumor responses among the 27 study patients, resulting in sustained and durable tumor regressions in 5 of them, all with either stage IIIc or M1a metastatic melanoma. Two of these responses were among the 6 patients enrolled in the NRA study that included both tremelimumab (one at 10 mg/kg and the other at 15 mg/kg, in both cases administered every 3 months) and the MART-1_26–35 _peptide pulsed DC vaccine. The other 3 patients with an objective response were among the 21 patients enrolled in the GA study administering single agent tremelimumab at 15 mg/kg every 3 months. For this study we graded toxicities during the first 3 months of therapy, which is considered one cycle. Among these patients there were 3 with toxicities that met the definition of DLTs as included in the clinical trial protocols, all in the GA study. These included two cases of grade 3 diarrhea or colitis and one patient with symptomatic panhypopituitarism (grade 2 hypophysitis). None of these patients received corticosteroids before the post-dosing apheresis.

**Table 1:** Patient characteristics

Patient ID	Sex	Age	Stage	Location of Metastasis	Treme-limumab (mg/kg q3mo)	MART-1/DC	Toxicities During the First Cycle	Tumor Response
NRA11	M	57	M1c	LN, Muscle	10	Y	-	PD

NRA12	M	55	M1c	Lung, Liver	10	Y	-	PD

NRA13	F	34	M1c	SC, LN, Muscle, Breast	10	Y	-	PD

NRA14	M	57	IIIc	SC	15	Y	-	CR

NRA15	M	48	M1a	LN	15	Y	-	PR

NRA16	F	61	M1a	S.C.	15	Y	-	PD

GA 5	M	65	M1c	Skin, LN, Adrenal	15	N	-	PR, then PD

GA 7	M	62	IIIc	Skin	15	N	G2 Pruritus	PD

GA 8	F	48	M1c	SC	15	N	G2 Diarrhea	PD

GA 9	M	52	M1c	LN, Bone	15	N	-	PD

GA 11	M	47	M1c	LN	15	N	-	PD

GA 12	M	76	M1c	Skin	15	N	G3 Colitis	PD

GA 13	M	37	M1a	LN	15	N	G2 Hypophysitis	PD

GA 14	M	38	M1c	SC, Muscle	15	N	-	PD

GA 15	M	58	M1c	Brain, Bowel, Liver	15	N	-	PD

GA 18	F	49	M1a	Skin	15	N	-	CR

GA 19	M	55	M1c	LN, Brain	15	N	G2 Diarrhea	PD

GA 21	M	71	M1c	Skin, SC, LN, Liver, Spleen	15	N	-	PD

GA 23	M	27	M1b	Lung	15	N	-	PD

GA 24	M	81	M1c	SC, Lung	15	N	-	PD

GA 25	M	71	M1c	LN	15	N	-	PD

GA 26	M	68	M1b	LN, Lung	15	N	G3 Diarrhea	PD

GA 27	M	52	M1c	SC	15	N	G2 Pruritus	PD

GA 28	M	48	M1c	LN, Lung	15	N	-	PD

GA 29	F	79	IIIc	Skin, SC	15	N	G2 Diarrhea	CR

GA 32	M	36	M1c	Muscle	15	N	-	PD

GA 33	F	49	IIIc	Skin	15	N	-	CR

MART-1/DC: MART-1_26–35 _peptide pulsed dendritic cells; G: grade; LN: lymph node; SC: subcutaneous; M: male; F: female; Y: yes; N: no; PD: progressive disease; SD: stable disease; PR: partial response; CR: complete response.

### No Change in IL-17 in Plasma of Patients Receiving Tremelimumab

**Figure 1:**
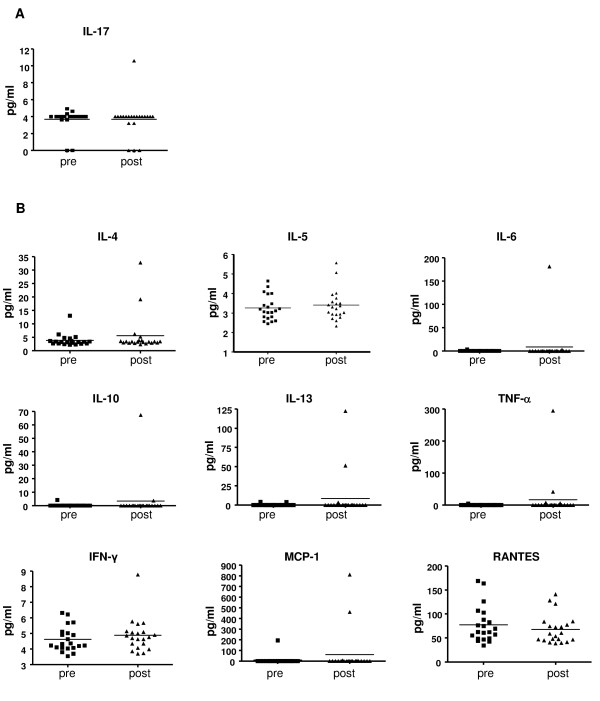
**Cytokine quantitation in patient's plasma**. *A*) ELISA analysis of IL-17 in cryopreserved plasma samples taken from patients before and after tremelimumab dosing. *B*) Multicytokine array quantifying IL-4, IL-5, IL-6, IL-10, IL-13, TNFα,, INF-γ, MCP-1 and RANTES in cryopreserved plasma before and after dosing with tremelimumab.

We analyzed the amount of IL-17 at baseline compared to post-tremelimumab aliquots of cryopreserved plasma obtained by apheresis. The concentration was very low in all samples (median of 4 pg/ml), and there were no evident differences between pre- and post-dosing samples (Figure 1A). We then analyzed an extended panel of cytokines in the same plasma samples using a multicytokine array to determine if a preferential cytokine profile was evident after CTLA4 blockade in patients. Levels of IL1-β, IL-2 and IL-12 were under the limit of detection for all samples. Levels of IL-4, IL-5, IL-6, IL-10, IL-13, TNF-α, INF-γ, MCP-1 and RANTES were detectable above the assay background, with no differences between pre- and post-dosing samples in most patients resulting in non-significant differences using a paired t test (Figure 1B). However, the results of one of the patients, GA18, are worth noting as an outlier in this group of patients. This patient entered the study with in transit skin metastasis that progressed after adjuvant interferon alpha 2b and GM-CSF, this last treatment stopped approximately two months before initiating tremelimumab. This patient went onto have a complete response that is ongoing over 1 year from study initiation. Table 2 provides complete results of the cytokine analysis in this patient, which demonstrates post-dosing increases in IL-4, IL-6, IL-10, IL-13, TNF-α, MCP-1 and RANTES (but not IL-5, IL-17 and INF-γ). These changes were not noted in any of the other 5 patients with an objective tumor response in this series, nor in patients with clinically-significant toxicities. In conclusion, there were no significant changes in circulating levels of cytokines after the administration of tremelimumab in most patients included in this series, and in particular there were no significant changes in circulating levels of IL-17 in the plasma of any patient.

**Table 2:** Cytokine levels in plasma of patient GA18

	Pre-dosing	Post-dosing
IL-4	3.31	32.78

IL-5	3.11	5.56

IL-6	0	181.45

IL-10	0	67.26

IL-13	0	122.46

IL-17	4.34	4

TNF-α	0	294.85

INF-γ	4.32	5.77

MCP-1	0	811.45

RANTES	102.67	141.16

### IL-17 Production Increases in *Ex Vivo *Activated PBMC

**Figure 2:**
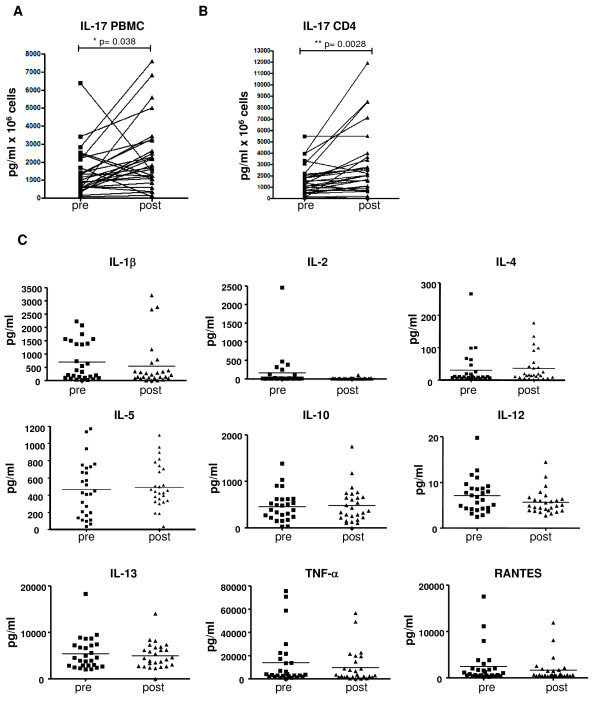
**IL-17 quantification by ELISA**. A and B) Pre- and post-dosing IL-17 cytokine determined in culture supernatants of whole PBMC (*A*) or CD4+-sorted cells (*B*) after stimulation for 4 days with anti-CD3 and anti-CD28. The supernatant was collected for IL-17 quantitation using an ELISA assay (p values by pairwise t-test). C) Multicytokine array in the same *ex vivo *stimulated samples quantifying IL-1β, IL-2, IL-4, IL-5, IL-10, IL-12(p70), IL-13, TNFα, and RANTES.

We examined the difference in the amount of IL-17 cytokine secreted by *ex vivo *activated cells obtained from pre- and post-dosing leukapheresis. The spontaneous cytokine production of non-stimulated PBMC was under the limit of detection for IL-17, as was for the rest of the cytokines measured by array (data not shown). Therefore, pre- and post-treatment whole PBMC and CD4-sorted cells were non-specifically stimulated with anti-CD3/anti-CD28 for 4 days and then analyzed for the amount of IL-17 in the culture supernatants by ELISA. IL-17 levels were significantly increased in the post-treatment samples as compared to the pre-treatment samples, with a similar profile in both supernatants from whole PBMC (Figure 2A) and magnetic column-sorted CD4 cells (Figure 2B). The culture supernatants from activated whole PBMC were also analyzed for an extended panel of cytokines by muticytokine array (Figure 2C). There were no differences in the concentrations of IL-1β, IL-2, IL-4, IL-5, IL-10, IL-13, TNF-α, and RANTES between pre- and post-dosing cultures. However, there was a significant decrease in IL-12(p70) in activated PBMC obtained after the administration of tremelimumab as compared to the secretion of this cytokine in activated baseline samples. Taken together, these data suggests a preferential increase in IL-17 production post-dosing.

### Th17 Cells Increase after CTLA4 Blockade

**Figure 3:**
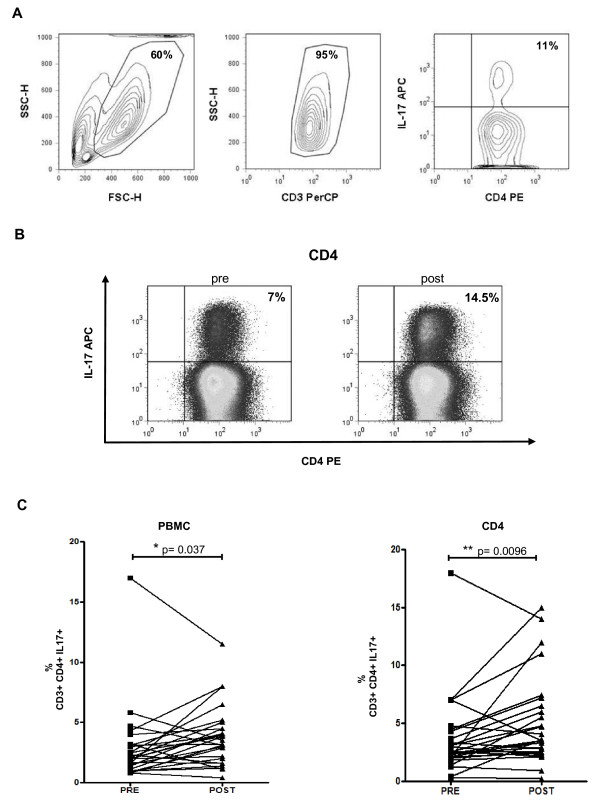
**Increase in Th17 cells after tremelimumab-based therapy by intracellular cytokine staining**. *A*) Gating strategy for IL-17 intracellular staining. Starting from either whole PBMC or CD-4 sorted cells (as depicted here), the lymphocyte population was gated on by FSC-H and SSC-H dot plot. Live cells were gated in the same graphic. A second gate was performed in CD3 and SSC-H dot plot. We analyzed for IL-17-producing cells among CD4+ T cells after gating. *B*) Example of IL-17 intracellular staining. After 4-day activation of CD4-sorted cells with anti-CD3 and anti-CD28, cells were additionally stimulated with PMA and ionomycin while inhibiting protein transport, and the number of Th17 cells was determined by flow cytometry. Depicted are the plots of gated Th17 cells from patient NRA12. The left column is the baseline pre-dosing sample, and the right column the post-dosing sample. *C*) Th17 quantification by flow cytometry. Pre- and post-dosing whole PBMC (left graph) or CD4+ cells (right graph) analyzed by flow cytometry for Th17 cells as described above (p values by pairwise t-test).

The number of IL-17-producing cells was analyzed by ICS after *ex vivo *stimulation of whole PBMC and isolated CD4 cells with anti-CD3/anti-CD28 for 4 days. To capture intracellular IL-17, these cultures were additionally stimulated for 5 hours with mitogens while cytokine secretion was inhibited with a protein transport inhibitor (see Materials and Methods). The lymphocyte population was gated first by morphology, followed by detection of T cells by anti-CD3 staining, and then Th17 quantitation as double positive CD4 cells with intracellular IL-17. Representative flow cytometric plots from one patient (NRA12) using CD4-sorted and stimulated cells (Figure 3A) demonstrate the increase in the population of Th17 cells when comparing pre- and post-dosing samples (Figure 3B). Double staining with anti-IL-17 and anti-CD4 antibodies in the samples from GA and NRA study patients revealed a statistically significant increase in the number of Th17 cells after tremelimumab treatment both in whole PBMC and in isolated CD4 cells (Figure 3C). Similar results were obtained when calculating the change in Th17 cells as an absolute number as opposed to a proportion (pre-dosing mean of 73,711 with 95% confidence interval of 46,912–100,510, compared with post-dosing mean of 101,066 with 95% confidence interval of 70,644–131,488, p = 0.026). We also analyzed the background values of IL-17 positive cells among unstimulated CD4+ cells. As expected, these values are low, with mean of 0.46 pre-dosing (95% confidence interval 0.22–0.7) and 0.62 post-dosing (95% confidence interval 0.49–0.75), with a trend (p = 0.15) in favor of increase in the post-dosing samples. Taken together with the cytokine profile in the culture supernatants, we conclude that there is a reproducible increase in IL-17-producing cells among activated blood cells after the administration of tremelimumab, suggesting an increase in Th17 cells with CTLA4 blockade in patients with metastatic melanoma.

### Preferential Increase in Th17 Cells in Patients with Autoimmune Toxicity after CTLA4 Blockade

**Figure 4:**
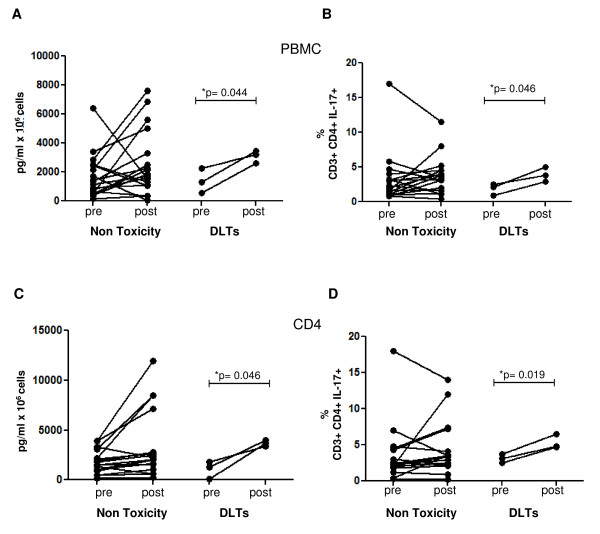
**IL-17 Intracellular Staining and IL-17 ELISA According to the Development of Inflammatory or Autoimmune Toxicity**. A and B) IL-17 secretion detected by ELISA as described in Figure 1, and Th17 by intracellular staining (ICS) as described in Figure 2, comparing the assay results in whole PBMC cultures from patients with Grade 3 or higher toxicity and the rest of the patients (p values by pairwise t-test). C and D) The same analysis with CD4-sorted cultures.

Since Th17 cells have been associated with inflammation, autoimmunity and antitumor responses, we explored the changes in pre- and post-dosing levels of IL-17-producing cells among patients with toxicity or response to tremelimumab-based therapy. There were no differences between samples from patients with or without an objective tumor response, either analyzed by IL-17 secretion in culture supernatants or by ICS for CD4 cells producing IL-17 (data not shown). Similarly, there were no differences between samples from the GA study administering tremelimumab alone and the NRA study where patients received both tremelimumab and an autologous DC vaccine. We then analyzed samples from patients with clinically significant toxicities during the first cycle of tremelimumab-based therapy (within 3 months from first dosing), meeting the prospectively-defined criteria for DLTs in these two studies described in the filing of the Investigator New Drug (IND) applications with the US Food and Drug Administration. This analysis demonstrated that the increase in Th17 cells is driven mostly by patients with toxicities. In PBMC from patients with toxicities the IL-17 increases were 2.3 and 2.2 fold when comparing pre- and post-dosing samples by ELISA and ICS, respectively, while in PBMC from patients without toxicity the respective increments were 1.5 and 1.1 fold. IL-17 increment in sorted CD4+ cells was 3.4 and 1.7 fold in patients with toxicity measured by ELISA and ICS, respectively, and 1.8 and 1.2 fold in PBMC from patients without toxicity. Even though the number of patients with toxicities is small in this series, the increase in IL-17-producing cells in patients with significant toxicities was highly reproducible, since it was evident and statistically significant when comparing IL-17 cytokine production in culture supernatants of activated whole PBMC and CD4-sorted cells (Figure 4A and 4B), as well as in the number of IL-17-producing cells determined by ICS, in both whole PBMC and CD4-sorted cells (Figure 4C and 4D).

## Discussion

Dose-escalation studies with CTLA4 blocking monoclonal antibodies provide clear evidence that increasing the antibody dose and exposure results in increasing toxicities consistent with breaking tolerance to self tissues, and at higher dosing levels, some patients benefit with durable tumor regressions [4,19,24]. Understanding the mechanism of both phenomena is of critical importance for this class of agents. It seems highly unlikely that the lymphocytes that mediate melanoma antitumor responses are the same as the ones that mediate toxicities like colitis, hypophysitis or thyroiditis, since there is little evidence of shared antigen profiles recognized by effector T cells among these tissues. Therefore, many studies have focused on studying immune cell subsets that are implicated in maintenance of peripheral tolerance. In particular, a lot of effort has been focused on detecting if Treg are decreased or functionally impaired in patients receiving CTLA4 blocking monoclonal antibodies. The interest is based on several lines of evidence, including the overlapping phenotype of autoimmune conditions in CTLA4 and FoxP3 deficient mice, and evidence that Treg-specific deficiency in CTLA4 expression impairs the suppressive function of Tregs [25]. The relatively high basal level of CTLA4 on Treg compared to activated T effector cells (which is the prime target for these blocking antibodies), and the clinical evidence of the modulation of peripheral tolerance with CTLA4 blocking antibodies, provided grounds for studying the implication of Treg in patient-derived samples. Most data reported to date demonstrate that the number of circulating cells with a Treg phenotype (CD4, CD25, FoxP3 positive) does not decrease after the administration of CTLA4 antibodies. In fact, there is a clear trend towards an increase in these cells [26-29], a finding that is not that surprising taking into account that these antibodies are blocking but not depleting antibodies for CTLA4 positive cells. Also, the number of cells staining positive for FoxP3 by immunohistochemistry increases in tumor biopsies of regressing lesions after CTLA4 blockade [20]. Data on functional modulation of Treg is not that clear, with mixed results on the detection of Treg-mediated suppression of effector T cells [26,28,29].

An alternative possibility studied by us is that Th17 cells, an immune cell subset implicated in mediating autoimmunity and in chronic inflammatory conditions, may be modulated by CTLA4 blocking antibodies. There is a reciprocal negative correlation between Treg and Th17 mediated by IL-2 [30], suggesting that their effects may be mutually exclusive as opposed to redundant. There is evidence that CTLA4 is expressed on murine Th17 cells at levels that are higher than Th1 cells [31], while CTLA4 has also been demonstrated on human Th17 cells [32]. Since both tremelimumab and ipilimumab, the two CTLA4 blocking antibodies in clinical development, inhibit CTLA4 negative signaling without inducing antibody-dependent cellular cytotoxicity (ADCC) [18,33], it is certainly possible that these antibodies would release negative signaling in Th17 resulting in increased number or function. In this study we analyzed IL-17 cytokine and cytokine-producing cells in peripheral blood of patients treated with tremelimumab with the goal of exploring if Th17 may be involved in the clinical events in patients receiving CTLA4 blocking monoclonal antibodies. Our data provides preliminary evidence that this may be the case. The modulation of Th17 levels is not large in magnitude, but is was highly reproducible among different assay conditions. Although we could not detect differences in IL-17 cytokine levels after dosing in plasma samples obtained directly from peripheral blood, the cells that had ability to produce IL-17 upon non-specific *ex vivo *stimulation increased in post-dosing blood cell samples from patients. This could be detected by quantifying soluble cytokine in culture supernatants and by determining the number of cells with intracellular IL-17 by flow cytometry. In addition, the results were comparable when we analyzed cultures from whole PBMC (including many immune and non-immune cell subsets other than CD4 T helper cells) and with sorted populations containing CD4 cells alone.

Th17 may be implicated in toxicities as well as responses after administration of anti-CTLA4 antibodies. Besides the well recognized implication of Th17 in murine and human inflammatory and autoimmune conditions [8], it is becoming clearer that they may also have a role in mediating antitumor immunity [17]. Therefore, we explored if the increases in Th17 cells were more prominent in the subsets of patients with toxicity or tumor responses. Although we found no correlation between IL-17 production and responses to therapy, our exploratory analysis suggests that the post-dosing increase in the levels of IL-17 in culture supernatants and by intracellular flow cytometry were higher in the small number of patients with toxicity. For this analysis, we restricted to clinically-significant toxicities that followed the prospective definition of DLTs in the clinical trial protocols, and which happened during the first cycle of therapy, the closest time to the obtaining of post-dosing samples in these patients. When samples from these patients were analyzed separately from samples from patients with lower levels of toxicity or no toxicities, differences between pre- and post-dosing samples were only evident in samples from patients with DLTs. The significance of increases in Th17 disappeared from the group of patients with non-DLT toxicities. Of note, patients with the highest levels of Th17 cells were not the ones who developed toxicities, suggesting to us that it is a doubling of the number of Th17 after tremelimumab may be linked to toxicities as opposed to the absolute number at any given time point. Our exploratory analysis is obviously limited by the small number of patients in this series, and will need to be confirmed in larger groups. However, the findings are reproducible in all of the different experimental conditions used to analyze IL-17-producing cells, which provides confidence in these results. From this work we conclude that Th17 may be implicated in the clinical effects of CTLA4 blocking monoclonal antibodies, and further study of their role in treatment-induced toxicities may help in elucidating how toxicities and responses may be differentially modulated with this mode of therapy.

## Competing interests

AR has received research funding and honoraria from Pfizer. The other authors have no competing interests on this work.

## Authors' contributions

EVE and AR conceived and designed the study. EVE, TC and NA carried out the experiments. JJ and BC-A provided the human samples for analysis. RCK and BC-A contributed to the assay conduct and data interpretation. EVE and AR wrote the manuscript. All authors read and approved the final manuscript.
